# Intermittent Hypoxic Exposure with High Dose of Arginine Impact on Circulating Mediators of Tissue Regeneration

**DOI:** 10.3390/nu12071933

**Published:** 2020-06-29

**Authors:** Agnieszka Zembron-Lacny, Artur Gramacki, Edyta Wawrzyniak-Gramacka, Anna Tylutka, Natalia Hertmanowska, Anna Kasperska, Miłosz Czuba

**Affiliations:** 1 Department of Applied and Clinical Physiology, Collegium Medicum University of Zielona Gora, 65-417 Zielona Gora, Poland; a.zembron-lacny@cm.uz.zgora.pl (A.Z.-L.); e.gramacka@cm.uz.zgora.pl (E.W.-G.); a.tylutka@cm.uz.zgora.pl (A.T.); n.hertmanowska@cm.uz.zgora.pl (N.H.); 2 Faculty of Computer, Electrical and Control Engineering. Institute of Control and Computation Engineering University of Zielona Gora, 65-417 Zielona Gora, Poland; a.gramacki@issi.uz.zgora.pl; 3 Department of Physiology, University School of Physical Education in Poznan, 61-871 Poznan, Poland; a.kasperska@gmail.com; 4 Department of Kinesiology, Institute of Sport, 01-982 Warsaw, Poland

**Keywords:** nitric oxide, hydrogen peroxide, growth factors, muscle damage, athletes

## Abstract

Intermittent exposure to hypoxia (IHE) increases production of reactive oxygen and nitrogen species which, as signalling molecules, participate in tissue injury–repair–regeneration cascade. The process is also stimulated by arginine whose bioavailability is a limiting factor for NO synthesis. The effects of IHE in combination with arginine (Arg) intake on myogenesis and angiogenesis mediators were examined in a randomized and placebo-controlled trial. Blood samples were collected from 38 elite athletes on the 1st, 7th and 14th days during the training camp. The oral doses of arginine (2 × 6 g/day) and/or IHE using hypoxicator GO2Altitude (IHE and Arg/IHE) were applied. Serum NO and H_2_O_2_ concentrations increased significantly and were related to muscle damage (CK activity >900 IU/mL) in IHE and Arg/IHE compared to placebo. The changes in NO and H_2_O_2_ elevated the levels of circulating growth factors such as HGF, IHG-1, PDGF^BB^, BDNF, VEGF and EPO. Modification of the lipid profile, especially reduced non-HDL, was an additional beneficial effect of hypoxic exposure with arginine intake. Intermittent hypoxic exposure combined with high-dose arginine intake was demonstrated to affect circulating mediators of injury–repair–regeneration. Therefore, a combination of IHE and arginine seems to be a potential therapeutic and non-pharmacological method to modulate the myogenesis and angiogenesis in elite athletes.

## 1. Introduction

The proliferation of satellite cells and vascularization are the essential processes in the regeneration of injured skeletal muscles. Myogenesis and angiogenesis are a prerequisite for the subsequent morphological and functional healing of the injured muscle. This, in turn, leads to rebuilding of the damaged myocytes and vessels, restoration of the blood flow and oxygen supply to the tissue. Nitric oxide (NO) plays a key role in repair response by inducing gene expression for several growth factors such as FGF, VEGF, IGF-1, HGF, PDGF^BB^ and BDNF, which are extracellular signals regulating the functions of the muscular, vascular and nervous systems. NO is produced from L-arginine by three isoenzymes called nitric oxide synthases (NOS), all present in skeletal muscles. While neuronal NOS (nNOS) and endothelial NOS (eNOS) are isoforms expressed constitutively, inducible NOS (iNOS) is mainly expressed during inflammatory response. NO generation can be modulated by intense physical training (physiological hypoxia), altitude training or training in hypoxic conditions, NO donors or NO precursors such as L-arginine [1,2,3].

Over recent years, intermittent hypoxic exposure (IHE) has been introduced into sport. IHE is a method by which athletes are exposed to short bouts of severe hypoxia (9–12% O_2_), interspersed with periods of normal air. Available studies reported substantial improvement in sea level endurance and anaerobic performance after IHE [4,5,6]. Beside the effects that hypoxia exerts on physical performance, there is some evidence that IHE might be beneficial for vascular endothelial activity and muscle regenerative capacity [7,8]. Hypoxic exposure increases production of reactive nitrogen and oxygen species (NO and H_2_O_2_) and affects metabolic pathways including mitochondrial respiration and biogenesis, apoptosis and, what has more recently been demonstrated, satellite cells proliferation. However, NO and H_2_O_2_ play a contradictory role in muscle regeneration and repair, i.e., in combination with growth factors, they lead to recovery of tissue function, whereas the local persistence of NO and H_2_O_2_ sustained by infiltrated neutrophils may cause further oxidative injury to differentiating myoblasts and myotubes, thereby delaying the complete health restoration [9,10]. So far, little is known on hypoxic exposure in combination with high arginine intake and their influence on the muscle injury, repair and regeneration. The typical dietary intake of L-arginine is set at approx. 3–8 g per day. Extracellular L-arginine can be rapidly taken up by endothelial cells and oxidized to NO. In a few clinical trials, intravenous (single dose of 30 g within 30 min) or dietary administration of relatively large doses of L-arginine (15–16 g per day) has been shown to result in enhanced NO formation, especially in subjects with endothelial dysfunction [11]. L-arginine also participates in other metabolic pathways, which are independent of NO synthesis but essential for physical performance. For instance, as a potent hormone secretagogue, L-arginine increases plasma levels of insulin, glucagon, growth hormone, insulin-like growth factor 1 and catecholamines [12]. Additionally, L-arginine may be metabolized via arginase whose high concentrations are identified in healing wounds due to macrophage production. Arginase activity results in ornithine formation, which is a precursor for proline that serves as substrate for collagen synthesis. Therefore, arginine supplementation could have a multidirectional impact on tissue regenerative processes [13,14]. On the basis of the gathered data, the present study was designed to explain whether hypoxic-induced NO and H_2_O_2_ generation contributes to the release of oxi-inflammatory mediators regulating the injury–repair–regeneration of skeletal muscles and to check whether high-dose L-arginine intake enhances NO and growth factors production during intermittent hypoxic exposure.

## 2. Materials and Methods

### 2.1. Subjects

Forty elite male wrestlers, members of the national team, were observed during preparatory periods for the new competition season (endurance training 53%, directed training 9% and special power training 38%). Each athlete underwent a thorough screening, including a full medical evaluation in National Centre for Sports Medicine. A two-week washout period was introduced before the training camp to avoid any possible interference of other nutritional supplements in the measured biochemical markers. Exclusion criteria included serious orthopaedic injury (*n* = 3), nutrition supplements or medications (*n* = 2), dehydration (*n* = 2) and anaemia (*n* = 1) identified at any point of the entire observation. Eventually, thirty-two athletes met all the criteria and completed the whole experiment (Table 1). The athletes participated in a 14-day training camp at the National Olympic Sport Centre. Prior to the training camp, the athletes were randomly assigned in a double-blind manner to a control group (placebo; methylcellulose capsules: 2 × 6 g per day for 12 days), an arginine group (Arg; capsules: 2 × 6 g per day for 12 days), a hypoxia group (IHE) and an arginine with hypoxic exposure group (Arg/IHE).

Throughout the camp, all athletes lived at the same accommodation and followed the same training schedule, sleeping time and diet. Daily energy value of food offered on the menu did not exceed 5200 kcal, and the protein dose varied from 1.6 to 1.8 g/kg of body mass. During the camp, the wrestlers consumed an isotonic sports drink Vitargo (osmolality 317 mOsm/kg H_2_O) or plain water. The dehydration level was assessed by Osmocheck calibrated in mOsm/kg H_2_O from 0 to 1500 mOsmols. All the subjects were informed of the aim of the study and signed a written consent to participate in the project. The protocol of the study was approved by the ethics committee at Medical University Poznan (N^o^550/11), in accordance with the Helsinki Declaration.

### 2.2. Arginine Supplementation

**Figure 1:**
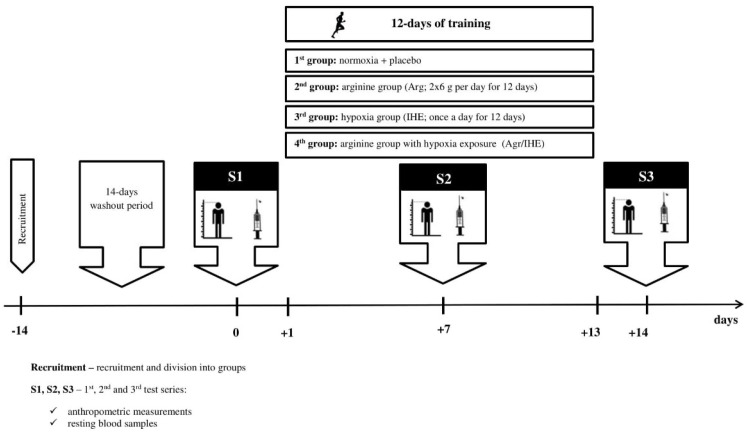
Illustration of the study design.

The flavour and appearance of arginine capsules (6 g capsules administered twice a day for 12 days) and placebo (methylcellulose capsules: 2 × 6 g per day for 12 days) were indistinguishable for both the subjects and the investigators. The subjects were instructed to consume their supplement 1 h before the morning training and 1 h before the afternoon training for 12 days of the training camp (Figure 1). Arginine and placebo capsules were prepared by Nutrend (The Czech Republic).

### 2.3. Intermittent Hypoxic Exposure

The passive 12-day intermittent hypoxic exposure (IHE) was conducted under medical supervision according to the procedure by Hinickson et al. [15] using the GO_2_Altitude hypoxicator (Australia) at the Olympic Sports Centre (Figure 1). The hypoxicator was able to simulate the height of 2500 to 6500 m above sea level by regulating oxygen levels at FiO_2_ of 14–9% (fraction of inspired oxygen). Intermittent hypoxic exposure parameters were determined after the preliminary assessment of athlete’s ability to adapt to hypoxic gas mixtures. The hypoxic test included the measurements of the time for the blood saturation to drop to 85% with FiO_2_ = 12% (equivalent to 4500 m above sea level) and the time for the blood saturation to return to 95% in normoxia. Based on the hypoxic test results, the IHE protocol was determined for every athlete. IHE was applied once a day, at least 2 h after sports training. Each IHE session consisted of 6 doses of 3–8-min periods of hypoxia (at FiO_2_ of 14–12%) interrupted by 3–5-min periods of normoxia, and repeated for 60–80 min. Hypoxic exposure started with two sessions with the oxygen concentration in the mask at FiO_2_ of 13.5% (equivalent to ~3000 m above sea level) and then reduced the oxygen concentration to FiO_2_ of 12% (equivalent to ~4500 m above sea level). The blood saturation (SpO_2_) and heart rate (HR) were individually monitored during every IHE session. SpO_2_ oscillated from 90.8 ± 2.4% on 1st day to 91.4 ± 5.6% whereas HR oscillated from 76.0 ± 7.8 bpm on 1st day to 76.1 ± 11.8 bpm on in groups exposed to hypoxia.

### 2.4. Body Composition

Body mass (BM) and body composition: fat-free mass (FFM) and fat mass (FM) were estimated using Tanita Body Composition Analyser MC-418 (Japan) calibrated prior to each test session in accordance with the manufacturer’s guidelines. Duplicate measures were taken with the participant in a standing position; the average value was used for the final analysis. The recurrence of measurement amounted to 98%. The measurements were taken between 7.00 and 8.00 a.m. before blood sampling.

### 2.5. Blood Sampling

Blood samples were taken 3-fold (on the 1st, 7th and 14th days of the training camp) from the median cubital vein between 7.00 and 8.00 a.m. after an overnight sleep, using S-Monovette tubes (Sarstedt, Austria). Within 20 min., they were centrifuged at 3000× *g* and +8 °C for 10 min. Aliquots of serum were stored at −80 °C. All samples were analysed in duplicate or triplicate in a single assay to avoid inter-assay variability. The intra-assay coefficients of variation (CV) for the used kits were <5%.

### 2.6. Skeletal Muscle Damage

Serum total creatine kinase (CK) activity was used as a marker of sarcolemma disruption and was evaluated by using commercially available reagents and mobile spectrophotometer DP 310 Vario II (Germany) at a temperature of 20–25 °C. The CK activity has been measured immediately after serum collection for the consecutive days of the conditioning camp. Percentage of changes in CK activity (%CK) was calculated by comparing the initial value on the 1st day with peak activity on the 7th and the 14th days of the conditioning camp.

### 2.7. Oxi-Inflammatory Mediators

Serum nitric oxide (NO) and hydrogen peroxide (H_2_O_2_) were measured by enzyme immunoassay and colorimetric methods using the Oxis Research kits (USA). NO and H_2_O_2_ detection limits were estimated at 0.5 µmol/L and 6.25 µmol/L, respectively. C-reactive protein (CRP) concentration was determined using commercial kit from DRG International (USA) with the detection limit 0.001 mg/L.

### 2.8. Growth Factors

Serum hepatocyte growth factor (HGF), insulin-like growth factor (IGF-1), muscle isoform of platelet-derived growth factor (PDGF^BB^), vascular endothelial growth factor (VEGF) and brain-derived neutrophic factor (BDNF) were evaluated by R&D Systems ELISA kits (USA). Detection limits were estimated at 40 pg/mL, 0.026 ng/mL, 15 pg/mL, 9 pg/mL and 20 pg/mL, respectively.

### 2.9. Lipoprotein-Lipid Profile

Total cholesterol (TC), high-density lipoproteins (HDL) and low-density lipoproteins (LDL) as well as triglycerides (TG) were determined by professional laboratory company Diagnostyka (Poland, ISO 15189). The non-HDL cholesterol was calculated by subtracting HDL from total cholesterol concentration.

### 2.10. Haematological Variables

The haematological markers (HB, RBC, RET, HTC, MCV, MCH, MCHC, RDW) and white blood cells (WBC) were determined by Diagnostyka (Poland, ISO 15189). Erythropoietin (EPO) was determined by enzyme immunoassay methods using the R&D Systems kits (USA). The detection limit for EPO was estimated at 0.6 mlU/mL.

### 2.11. Statistical Analysis

Statistical analyses were performed using the R system, version 3.6.1 [https://www.r-project.org]. The significant differences in mean values between the groups (Control, Arg, IHE and Arg/IHE) were assessed mainly by the one-way ANOVA and the Tukey’s post hoc tests. The assumptions for the use of parametric or non-parametric tests were checked using the Shapiro–Wilk and the Levene tests to evaluate the normality of the distributions and the homogeneity of variances, respectively. The significant differences for NO and H_2_O_2_ were assessed first by the one-way MANOVA. A statistically significant one-way MANOVA can be followed up by univariate one-way ANOVA examining, separately, each dependent variable. Moreover, the appropriate multivariate tests for checking assumptions were used (i.e., multivariate normality and homogeneity of variance-covariance matrices). In the case of ANOVA, if the normality and homogeneity assumptions were violated, the Kruskal–Wallis non-parametric test was used. The comparisons of repeated measurements (1st vs. 7th and 1st vs. 14th days of the camp) were assessed by the t-Student test or the Wilcoxon signed-rank test depending on compliance with the normality assumption. Additionally, eta-squared (*η*^2^) was used as a measure of effect size, which is indicated as having no effect if 0 ≤ *η*^2^ < 0.05, a minimum effect if 0.05 ≤ *η*^2^ < 0.26, a moderate effect if 0.026 ≤ *η*^2^ < 0.64 and a strong effect if *η*^2^ ≥ 0.64 [16]. Pearson’s correlation coefficients were calculated to describe the relationships between CK, NO, H_2_O_2_ and growth factors. Statistical significance was set at *P* < 0.05.

## 3. Results

### 3.1. Skeletal Muscle Damage

CK activity reached 2-fold increase on the 7th day and 14th days of the conditioning camp in controls and Arg whereby %CK did not significantly differ between groups. The hypoxic exposure increased %CK activity significantly by 357 ± 78% on the 7th day and by 684 ± 117% on the 14th day in IHE and by 263 ± 159% on the 7th day and by 586 ± 270% on the 14th day compared to the 1st day of the training camp in Arg/IHE group. However, CK did not reach the values >3000 IU/L, which were observed following very intensive training in our previous study [17]. The total CK activity highly correlated with NO (r = 0.720, *P* < 0.001) and H2O2 (r = 0.646, *P* < 0.001). This indicates that hypoxia-induced generation of reactive oxygen and nitrogen species increases skeletal muscles damage.

### 3.2. Oxi-Inflammatory Mediators

**Figure 2:**
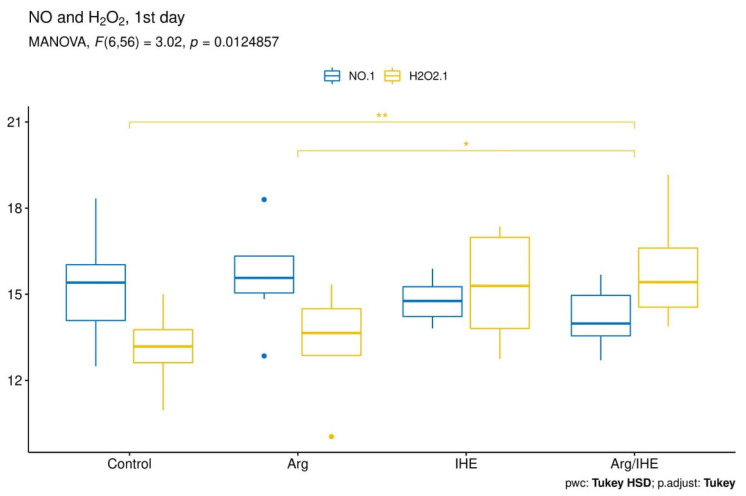
Visualisation of the MANOVA statistical analysis of changes in nitric oxide (NO) and hydrogen peroxide (H_2_O_2_) levels on the 1st day of the training camp for the following groups: control, arginine (Arg), hypoxic exposure (IHE) and arginine with hypoxic exposure (Arg/IHE). Results of the Tukey HSD post-hoc comparisons are coded as follows: * *P* < 0.05, ** *P* < 0.01. The homogeneity of covariances and multivariate normality assumptions are met.

**Figure 3:**
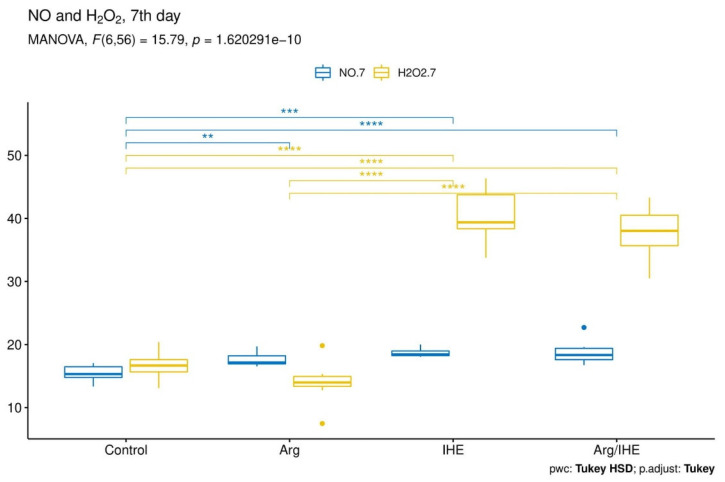
Visualisation of the MANOVA statistical analysis of changes in nitric oxide (NO) and hydrogen peroxide (H_2_O_2_) levels on the 7th day of the training camp for the following groups: control, arginine (Arg), hypoxic exposure (IHE) and arginine with hypoxic exposure (Arg/IHE). Results of the Tukey HSD post-hoc comparisons are coded as follows: ** *P* < 0.01, *** *P* < 0.001, **** *P* < 0.0001. The homogeneity of covariances and multivariate normality assumptions are met.

**Figure 4:**
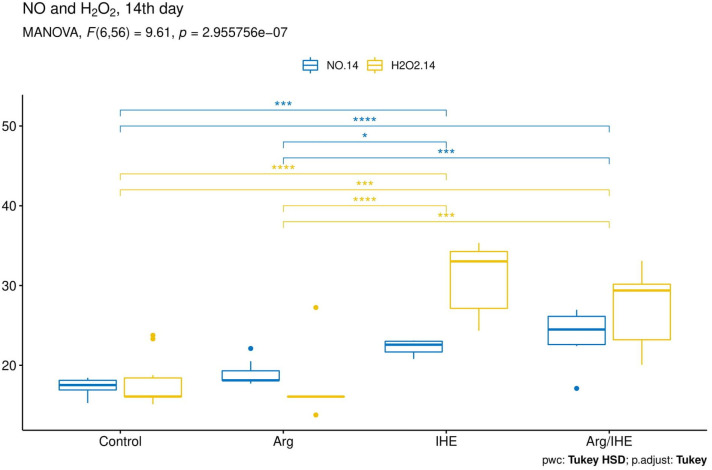
Visualisation of the MANOVA statistical analysis of changes in nitric oxide (NO) and hydrogen peroxide (H_2_O_2_) levels on the 14th day of the training camp for the following groups: control, arginine (Arg), hypoxic exposure (IHE) and arginine with hypoxic exposure (Arg/IHE). Results of the Tukey HSD post-hoc comparisons are coded as follows: * *P* < 0.05, *** *P* < 0.001, **** *P* < 0.0001. The homogeneity of covariances and multivariate normality assumptions are met.

The results are presented in Table 2. The changes in NO and H_2_O_2_ concentrations proceeded simultaneously and reached the highest values on the last day of the training camp (Figure 2, Figure 3 and Figure 4). The hypoxic exposure resulted in above 2-fold increase in NO and H_2_O_2_ concentration on the 7th day of the training camp, and during the following days NO and H_2_O_2_ remained at a high level in IHE and Arg/IHE groups. The NO/H_2_O_2_ ratio decreased in IHE and Arg/IHE compared to control group, which indicates a more significant influence of intermittent hypoxia on H_2_O_2_ than on NO generation. The value η^2^ showed a strong effect of arginine and/or IHE on NO and H_2_O_2_ concentrations on the 7th and 14th days of observation, i.e., the time of arginine and/or IHE administration determined the extent of changes in both molecules. CRP concentration significantly increased in response to hypoxic exposure in IHE and Arg/IHE compared with control on the 7th day, but it remained on the level <5 mg/L. Since the increased CRP level did not exceed the reference values, we concluded that hypoxic exposure applied in our study did not adversely affect the athletes’ inflammatory status.

### 3.3. Growth Factors

The results are presented in Table 3. The changes in circulating growth factors levels proceeded differently following sports training and arginine intake, and they were dependent on skeletal muscle damage. The levels of PDGFBB, BDNF and VEGF increased on the 7th day; HGF level rose on the 14th day whereas IGF-1 decreased in controls and Arg. Hypoxic exposure elevated growth factors except for BDNF, which significantly decreased on the 7th and 14th days compared to the initial level and control group. NO and H_2_O_2_ generation significantly modulated the release of growth factors into the circulation. The strongest association was observed for NO, H_2_O_2_ and IGF-1 (Table 4). The value η^2^ indicated a strong effect of arginine and/or IHE administration on growth factors concentrations, particularly on the 14th day of observation. This means that the duration of use of arginine and hypoxia determine the extent of the changes in growth factors, similarly to nitric oxide.

### 3.4. Haematological Variables

The results are presented in Table 5. No changes in HB concentration were observed whereas contrasting changes were identified in other haematological markers, i.e., RBC and HTC levels decreased while RET concentration increased in all subjects during the 14-day observation. The serum EPO level rose significantly in all subjects on the 7th and 14th days. However, what hypoxia exposure, alone or with arginine intake, exerted the greatest influence on was EPO concentration. EPO level highly correlated with NO (r = 0.624, *P* < 0.001) and H_2_O_2_ (r = 0.600, *P* < 0.001), which indicates their substantial share in erythropoietin synthesis. A moderate effect of arginine and/or IHE administration on EPO concentration was also proven by the value η^2^. WBC count increased on the 14th day of the training camp in all subjects but fell within the normal range (from 4.0 × 10^3^/µL to 10.0 × 10^3^/µL). Therefore, we concluded that arginine intake and/or hypoxic exposure applied in our study did not adversely affect the athletes’ immune function.

### 3.5. Lipoprotein–Lipid Profile

The results are presented in Table 6. TG, TC, LDL and HDL concentrations were found to be at similar levels in all subjects. On the 1st day of observation, high levels of TC and LDL were detected in 40% (>200 mg/dL) and 31% (>130 mg/dL) of the subjects, respectively. Finally, non-HDL exceeded the level of 145 mg/dL in 40% of the athletes. A decreasing trend of all the elements of lipid profile was observed on 14th day in Arg and IHE when compared to control group. Interestingly, hypoxic exposure induced approx. 20% decrease in non-HDL on the 7th and 14th days compared to the 1st day of sports training. The value η2 showed a moderate impact of arginine and/or IHE administration on non-HDL concentration, especially on the 14th day of the observation.

## 4. Discussion

Skeletal muscle regeneration is a complex event that includes changes in generation of reactive and oxygen species, interactions between skeletal muscle and the immune system as well as satellite cells activation [18]. In sports medicine, the efficiency of regenerative processes is decisive for athletes’ health and physical performance. Therefore, new therapies are being sought to modify the cascade of injury–repair–regeneration of skeletal muscles. In many cases, regeneration-stimulating methods are implemented into clinical medicine to improve functional abilities in patients awaiting surgical interventions or suffering from chronic illnesses such as cardiovascular and rheumatic diseases [4,8,13,19,20,21,22]. NO and H_2_O_2_ production is known to increase dramatically in injured skeletal muscle [18,23]. In addition, previous studies have shown that persistent inflammation after exercise-induced muscle damage is accompanied by reduced NO bioavailability and excessive H_2_O_2_ concentration [18,24,25,26]. In the present study, intensive sport training significantly elevated NO and H_2_O_2_ concentrations but lowered NO/H_2_O_2_ ratio, which indicates H_2_O_2_ overproduction compared to NO. Hypoxic exposure enhanced H_2_O_2_ concentration on the 7th day and NO level on the 14th day of training camp in IHE and Arg/IHE. A large number of studies have demonstrated that intermittent hypoxia increases the production of both molecules in a variety of model systems, including cells, blood vessels, muscles and isolated hearts [27,28,29,30,31]. Under conditions which amplify or prolong the initial inflammatory response, manifested by CRP increase, muscle damage can be considerably increased by NO and H_2_O_2_ produced in neutrophils and macrophages by inducible nitric oxide synthase (iNOS) and NADPH oxidase (NOX). Hypoxia enhances iNOS expression in pro-inflammatory macrophages M1 which, having reached their peak concentration in injured and regenerating muscle, are then replaced by a population of M2 macrophages that can attenuate the inflammatory response and promote tissue repair [18]. Some reports have suggested that endothelial NOS expression is similarly elevated in hypoxia-exposed animals [32]. In or study, skeletal muscle damage was the primary cause of NO and H_2_O_2_ release from active immune cells. The highest levels of NO and H_2_O_2_ were observed in subjects exposed to hypoxia and who demonstrated the highest CK activity >900 IU/L. This increase in NO and H_2_O_2_ levels strongly correlated with CK activity. Total CK activity is a widely used measurement in monitoring of the training load, physical efficacy and overtraining [17,33]. Morris et al. [34] demonstrated that muscle damage biomarkers increased over time and exceeded the normal reference ranges at moderate altitude (hyperbaric hypoxia), indicating cell damage pathology. Hypoxia impairs injured muscles rebuilding in patients with chronic obstructive pulmonary disease and peripheral arterial disease, but it has a positive effect on the muscle regenerative capacity in athletes [7,35]. The high dose of oral arginine, applied in our study, did not have any effect on NO and H_2_O_2_ production and muscle damage provoked by intensive training and hypoxic exposure. Similar observations have recently been reported by Alvares et al. [36], Forbes and Bell [37], Forbes et al. [38], Meirelles and Matsuura [39] and Meirelle et al. [40]. Studies in human isolated muscle and myotube culture have demonstrated that NO and H_2_O_2_ are key regulators of pre- and posttranslational signalling events leading to cytokines, heat-stress proteins and growth factors synthesis [41]. The growth factors especially involved in myogenesis include HGF, IGF-1, PDGF^BB^, VEGF and BDNF, which are released from leucocytes and muscle cells within a few hours after muscle damage and then secreted from other tissues during the following few days. The timing and availability of these growth factors, as well as their receptors density on or within the myogenic satellite cells, are critical mediators in the regenerative process [42]. In our study, the changes in circulating growth factors proceeded differently in time following sports training, arginine intake and/or hypoxic exposure; however, all the growth factors were found to be related to NO and H_2_O_2_ generation (Table 4). Hypoxic exposure was observed to elevate growth factors levels, except for BDNF, which significantly decreased on the 7th and 14th days of the training camp. The changes were more pronounced when arginine with IHE were used, especially for IGF-1 and VEGF. The value *η*^2^ indicated that arginine and/or IHE administration produced the strongest effect on IGF-1 in comparison with other growth factors. According to Oh et al. [12] arginine promotes the synthesis and secretion of IGF-1 from hepatocytes through the mitogen-activated protein kinases (MAPK) cascades that are central signalling pathways and regulate a wide variety of cellular processes, including proliferation, differentiation, apoptosis and stress response. The authors showed dual function of arginine in cellular processes. Firstly, arginine directly activated MAPK signalling cascades, as a short-term effect; secondly, it induced IGF-1 mRNA expression and subsequent secretion, as seen via a long-term treatment. Arginine transport into endothelial cells is augmented by VEGF, and this effect can be modulated by hypoxia [43,44]. VEGF improves skeletal muscle repair through modulation of angiogenesis; however, recent studies concerning therapeutic vascularization have demonstrated that the mechanism is regulated by PDGF^BB^ [45]. In our study, the highest levels of VEGF and PDGF^BB^ were observed in the group where simultaneous use of arginine and hypoxia exposure were applied.

The regenerative process requires multiple factors to work in a coordinated way in order to restore tissue metabolic functionality. In our study, changes in NO and H_2_O_2_ caused by simultaneous Arg and IHE application were demonstrated to be associated most significantly with circulating IGF-1 and HGF, and further on with PDGF^BB^ and BDNF (Table 4). HGF has been proven to increase myogenic satellite cells migration to the site of injury and to play a prominent role in regulation of early phases of muscle regeneration. Its release from the muscle extracellular matrix is initially mediated via NO release after mechanical or injury-induced signals [46]. The BDNF, in turn, is the circulating factor which deserves special attention. This growth factor is part of the neurotrophic family and is responsible for the viability and functioning of a variety of neuronal subtypes within the brain. In skeletal muscle, BDNF is accountable for proliferation and differentiation of satellite cells as well as growth of the myofibers [47]. The available data show that almost 70–80% of circulating BDNF come from the brain and 25% from contracting muscles [48,49]. In this study, a decrease in BDNF concentration following hypoxic exposure was observed at a similar level, approx. 40%, in both IHE and Arg/IHE groups. To date, the mechanism of hypoxia effect on the central nervous system has been poorly investigated. Some data suggest that hypoxic exposure reduces cognitive functions and modifies central motor command [50,51]. However, Piotrowicz et al. [52] recently demonstrated that neurotrophins, which are considered as brain damage markers, were not affected by hypoxic conditions, which proves IHE safety for the central nervous system.

NO and H_2_O_2_ are involved in signal transduction pathways as part of the O_2_-sensing mechanism stabilizing transcription factor HIF-1 and regulating erythropoietin expression. EPO regulates the process of haematopoiesis, stabilizes vascular integrity, increases the number of endothelial cells and protects these cells against ischemia and apoptosis [52,53]. According to Jia et al. [54] EPO contributes directly to myoblast proliferation and survival, leading to muscle regeneration and repair. The EPO expression during hypoxia is more dependent on changes in H_2_O_2_, whereas in non-hypoxic conditions it is related to NO, pro-inflammatory cytokines IL-β and TNFα as well as growth factors [55]. In this study, hypoxic exposure, alone or with arginine intake, was found to have a significant impact on serum EPO level in relation to H_2_O_2_ and NO during a 14-day observation. It has been described that HIF-1 transcriptional activity and EPO expression are achieved through two parallel mechanisms, i.e., a decrease in O_2_-dependent hydroxylation of HIF-1 and S-nitrosylation of HIF-1 pathway components [30]. Beleslin-Cokic et al. [56] provided evidence that hypoxia and EPO increased the endothelial cells capacity of NO production.

The mechanisms involved in the generation of reactive oxygen and nitrogen species are critical for endothelial function [30]. The NO and H_2_O_2_ overproduction as well as nitration of many proteins cause a decrease of enzyme activity, disrupt metabolism and cellular detoxification, impair cytoskeletal organization and ultimately contribute to the cytotoxic effects of peroxynitrite. Interestingly, in this study, arginine and/or hypoxic exposure was observed to induce a decrease in non-HDL by approx. 20% on the 7th and 14th days compared to the 1st day of sports training. Other elements of lipid profile tended to decrease when compared to controls. According to Bailey et al. [4] and Mallet et al. [57], intermittent hypoxic exposure increases cardiovascular resistance to ischemia-reperfusion stress and can be safely used clinically to protect subjects with developing coronary disease or those awaiting cardiac procedures. In sport, intermittent hypoxic exposure is commonly used to increase physical performance. However, our study demonstrated that IHE alone or in combination with arginine can simultaneously enhance regenerative capacity of skeletal muscle and protect athletes from endothelial dysfunction, especially the athletes participating in sports that include strength elements.

## 5. Conclusions

In this study we demonstrated for the first time that intermittent hypoxic exposure and high arginine intake collectively contribute to the release of mediators that regulate the injury–repair–regeneration of skeletal muscles and endothelium. Therefore, simultaneous application of IHE and arginine seems to have favourable and therapeutic potential to modulate the myogenesis and angiogenesis, especially in athletes undergoing strenuous training schedule. However, the transfer of hypoxic exposure with arginine intake to a clinical setting to enhance skeletal muscles repair or to reduce an endothelial dysfunction requires further studies.

## 6. Limitations

The limitations of the study include a relatively small number of subjects and no continuation of experiment after the training camp; however, it proved sufficient to show a protective effect of hypoxic exposure and arginine intake in elite athletes typically engaged in very high-intensity exercise training. Moreover, few epidemiologic and physiologic observations in athletes make it difficult to explain the impact of simultaneous application of IHE and arginine on the tissue regeneration.

## Figures and Tables

**Table 1:** Anthropometrics and body composition (mean ± SD).

	Control *n* = 10	Arg *n* = 7	IHE *n* = 6	Arg/IHE *n* = 9	Control vs. Arg IHE Arg/IHE
Age [yr.]	24.6 ± 3.0	20.0 ± 1.6	22.8 ± 2.6	24.7 ± 4.4	<0.05 0.622 0.999
Height [cm]	173.6 ± 8.8	179.0 ± 9.5	181.2 ± 7.3	175.6 ± 8.3	0.559 0.320 0.947
Weight [kg]	81.4 ± 21.8	79.9 ± 13.0	97.1 ± 22.7	87.9 ± 20.7	0.989 0.560 0.957
BMI [kg/m^2^]	26.6 ± 4.5	24.4 ± 1.4	29.3 ± 5.2	27.8 ± 4.5	0.552 0.773 0.990
%FM	18.1 ± 4.8	9.3 ± 3.0	14.5 ± 6.0	21.3 ± 6.5	<0.05 0.844 0.252
FM [kg]	15.4 ± 7.4	7.6 ± 3.2	15.1 ± 9.5	19.3 ± 10.8	0.355 0.998 0.569
FFM [kg]	66.0 ± 15.0	72.3 ± 10.6	81.9 ± 14.0	67.2 ± 11.5	0.952 0.270 0.996

**Abbreviations**: Arg, arginine intake; IHE, intermittent hypoxic exposure; Arg/IHE, arginine intaken and intermittent hypoxic exposure; BMI, body mass index; FM, fat mass; FFM, fat-free mass. The significant differences in mean values between the groups (Control vs. Arg, Control vs. IHE, Control vs. Arg/IHE were assessed by the one-way ANOVA and Tukey’s post hoc test.

**Table 2:** The levels of oxi-inflammatory mediators.

	1st Day of Camp			7th Day of Camp			14th Day of Camp			1st Day vs. 7th Day	1st Day vs. 14th Day
	mean ± SD	Control vs. Arg, IHE or Arg/IHE	*η* ^2^	mean ± SD	Control vs. Arg, IHE or Arg/IHE	*η* ^2^	mean ± SD	Control vs. Arg, IHE or Arg/IHE	*η* ^2^		
**NO** [µmol/L]											
Control	15.37 ± 1.83	-	0.166	15.36 ± 1.26	-	0.569	17.33 ± 0.99	-	0.701	0.995	<0.05
Arg	15.64 ± 1.66	0.98		17.67 ± 1.19	<0.01		18.97 ± 1.67	0.326		<0.01	<0.01
IHE	14.78 ± 0.79	0.856		18.71 ± 0.73	<0.001		22.26 ± 0.95	<0.001		<0.05	<0.001
Arg/IHE	14.12 ± 0.95	0.251		18.67 ± 1.78	<0.001		23.93 ± 3.02	<0.001		<0.001	<0.01
**H_2_O_2_** [µmol/L]											
Control	13.16 ± 1.13	-	0.382	16.67 ± 2.00	-	0.92	17.87 ± 3.15	-	0.678	<0.001	<0.01
Arg	13.39 ± 1.76	0.992		13.99 ± 3.66	0.46		17.34 ± 4.45	0.994		0.748	<0.05
IHE	15.26 ± 1.99	0.098		40.36 ± 4.64	<0.001		30.93 ± 4.81	<0.001		<0.001	<0.001
Arg/IHE	15.97 ± 1.91	<0.01		39.47 ± 4.36	<0.001		27.41 ± 4.63	<0.001		<0.001	<0.001
**NO/H_2_O_2_ ratio** [µmol/L]											
Control	1.17 ± 0.10	-	0.456	0.93 ± 0.13	-		0.99 ± 0.15	-	0.386	<0.01	<0.05
Arg	1.19 ± 0.23	0.986		1.35 ± 0.42	<0.01		1.13 ± 0.19	0.383		0.505	0.675
IHE	0.98 ± 0.13	0.095		0.47 ± 0.05	<0.01		0.74 ± 0.15	<0.05		<0.001	<0.05
Arg/IHE	0.90 ± 0.12	<0.01		0.51 ± 0.10	<0.01		0.90 ± 0.20	0.615		<0.001	0.974
**CRP** [mg/L]											
Control	1.57 ± 0.53	-	0.024	2.02 ± 0.35	-	0.552	2.29 ± 0.48	-	0.115	<0.05	<0.01
Arg	1.62 ± 0.49	0.997		1.99 ± 0.55	0.999		2.14 ± 0.41	0.917		0.182	0.097
IHE	1.45 ± 0.15	0.968		2.69 ± 0.41	<0.05		2.46 ± 0.28	0.913		<0.001	<0.001
Arg/IHE	1.66 ± 0.57	0.977		3.09 ± 0.52	<0.001		2.58 ± 0.63	0.589		<0.001	<0.01

**Abbreviations:** NO, nitric oxide; H2O2, hydrogen peroxide; CRP, C-reactive protein; η2 is a measure of effect size. Data in columns whose names begin with “Control” show the P-values of the Tukey’s post-hoc tests of the univariate one-way ANOVA examining, separately, each dependent variable. The last two columns show the P-values of the t-Student test or the Wilcoxon nonparametric test (if the normality assumption is violated).

**Table 3:** The levels of tissue regeneration mediators.

	1st Day of Camp			7th Day of Camp			14th Day of Camp			1st Day vs. 7th Day	1st Day vs. 14th Day
	mean ± SD	Control vs. Arg, IHE or Arg/IHE	*η* ^2^	mean ± SD	Control vs. Arg, IHE or Arg/IHE	*η* ^2^	mean ± SD	Control vs. Arg, IHE or Arg/IHE	*η* ^2^		
**HGF [pg/mL]**											
Control	587 ± 73	-	0.237	534 ± 77	-	0.467	602 ± 68	-	0.653	<0.01	0.625
Arg	620 ± 60	0.687		568 ± 89	0.827		770 ± 69	<0.001		<0.05	<0.01
IHE	623 ± 61	0.666		546 ± 95	0.992		792 ± 49	<0.001		0.094	<0.05
Arg/IHE	544 ± 42	0.425		708 ± 72	<0.001		789 ± 71	<0.001		<0.001	<0.001
**IGF-1** [ng/mL]											
Control	120 ± 41	-	0.19	116 ± 22	-	0.867	100 ± 15	-	0.911	0.16	0.084
Arg	126 ± 23	0.982		103 ± 19	0.834		98 ± 12	0.998		0.073	<0.01
IHE	149 ± 11	0.231		248 ± 26	<0.001		206 ± 29	<0.001		<0.001	<0.01
Arg/IHE	148 ± 26	0.186		280 ± 51	<0.001		293 ± 43	<0.001		<0.001	<0.001
**PDGFBB** [pg/mL]											
Control	2281 ± 513	-	0.084	2646 ± 289	-	0.381	2068 ± 368	-	0.706	0.074	0.063
Arg	2327 ± 418	0.995		2836 ± 293	0.806		1980 ± 161	0.927		0.062	0.088
IHE	2582 ± 231	0.456		2762 ± 160	0.953		3133 ± 263	<0.001		0.15	<0.01
Arg/IHE	2307 ± 266	0.999		3418 ± 686	<0.01		2478 ± 274	<0.05		<0.01	0.226
**BDNF** [pg/mL]											
Control	23,447 ± 3237	-	0.067	27,486 ± 1974	-	0.781	27,426 ± 2452	-	0.789	<0.05	<0.01
Arg	23,922 ± 3040	0.987		29,567 ± 2651	0.301		26,626 ± 1250	0.88		<0.05	0.073
IHE	22,402 ± 3184	0.899		18,817 ± 1118	<0.001		18,154 ± 1377	<0.001		0.059	0.053
Arg/IHE	22,120 ± 2177	0.756		21,218 ± 3025	<0.001		19,952 ± 2791	<0.001		0.198	<0.05
**VEGF** [pg/mL]											
Control	341 ± 68	-	0.03	405 ± 54	-	0.112	234 ± 65	-	0.782	0.085	<0.001
Arg	361 ± 64	0.861		408 ± 63	0.958		238 ± 77	0.999		0.154	<0.05
IHE	330 ± 44	0.999		406 ± 46	0.974		389 ± 45	<0.001		<0.05	0.135
Arg/IHE	344 ± 78	0.991		452 ± 97	0.284		495 ± 64	<0.001		0.054	<0.01

**Abbreviations**: Arg, arginine supplementation; IHE, intermittent hypoxic exposure; Arg/IHE, arginine supplementation and intermittent hypoxic exposure; HGF, hepatocyte growth factor; IGF-1, insulin-like growth factor 1β; PDGF^BB^, platelet-derived growth factor; BDNF, brain-derived neurotrophic factor; VEGF, vascular endothelial growth factor; η^2^ is a measure of effect size. Data in columns whose names begin with “Control” show the P-values of the Tukey’s post-hoc tests of the univariate one-way ANOVA examining, separately, each dependent variable. The last two columns show the P-values of the t-Student test or the Wilcoxon nonparametric test (if the normality assumption is violated).

**Table 4:** Relationships (Pearson’s correlation coefficients) between oxi-inflammatory mediators NO and H_2_O_2_, and growth factors HGF, IGF-1, PDGF^BB^, BDNF and VEGF.

	HGF [pg/mL]	IGF-1 [ng/mL]	PDGF^BB^ [pg/mL]	BDNF [pg/mL]	VEGF [pg/mL]
**NO** [µmol/L]	0.662	0.554	0.160	−0.286	0.274
	<0.001	<0.001	>0.05	<0.01	<0.01
**H_2_O_2_** [µmol/L]	0.321	0.780	0.479	−0.525	0.368
	<0.01	<0.001	<0.001	<0.001	<0.001

**Table 5:** Haematological markers and immune cells count.

	1st Day of Camp			7th Day of Camp			14th Day of Camp			1st Day vs. 7th Day	1st day vs. 14th day
	mean ± SD	Control vs. Arg, IHE or Arg/IHE	*η* ^2^	mean ± SD	Control vs. Arg, IHE or Arg/IHE	*η* ^2^	mean ± SD	Control vs. Arg, IHE or Arg/IHE	*η* ^2^		
**HB [g/dL]**											
Control	15.3 ± 0.8	-	0.043	15.2 ± 0.7	-	0.165	15.5 ± 0.2	-	0.462	0.918	0.335
Arg	14.9 ± 0.8	0.757		14.8 ± 0.5	0.617		14.6 ± 0.3	<0.01		0.271	0.306
IHE	15.1 ± 0.8	0.961		14.4 ± 1.0	0.137		14.3 ± 0.6	<0.01		0.071	0.218
Arg/IHE	15.3 ± 0.7	1		15.1 ± 0.7	0.947		15.2 ± 0.8	0.517		0.16	0.851
**RBC** [mln/mm^3^]											
Control	5.4 ± 0.3	-	0.189	5.4 ± 0.2	-	0.392	5.1 ± 0.2	-	0.736	0.411	<0.05
Arg	5.3 ± 0.5	0.781		5.2 ± 0.3	0.286		4.6 ± 0.1	<0.001		0.636	<0.05
IHE	5.2 ± 0.4	0.852		5.0 ± 0.5	<0.05		4.7 ± 0.3	<0.01		0.055	<0.05
Arg/IHE	5.0 ± 0.2	0.076		4.9 ± 0.2	<0.01		5.5 ± 0.3	<0.05		0.065	<0.01
**RET** [‰]											
Control	4.1 ± 1.1	-	0.222	5.2 ± 1.2	-	0.459	7.1 ± 2.2	-	0.505	<0.05	<0.01
Arg	4.1 ± 1.2	1		5.0 ± 1.4	0.994		10.1 ± 1.8	<0.05		0.2	<0.001
IHE	3.0 ± 0.6	0.139		8.7 ± 2.5	<0.001		12.0 ± 1.4	<0.001		<0.01	<0.001
Arg/IHE	3.3 ± 0.7	0.295		6.4 ± 1.1	0.319		9.3 ± 1.7	0.071		<0.001	<0.01
**HCT** [%]											
Control	48.1 ± 3.0	-	0.4	48.2 ± 2.8	-	0.45	45.8 ± 1.5	-	0.403	0.825	<0.05
Arg	47.9 ± 3.0	0.996		47.2 ± 1.8	0.878		43.0 ± 1.2	<0.01		0.444	<0.01
IHE	45.7 ± 1.1	0.221		45.1 ± 2.4	0.164		44.2 ± 2.4	0.176		0.598	0.096
Arg/IHE	43.8 ± 1.7	<0.01		42.3 ± 3.7	<0.001		45.8 ± 1.0	1		0.41	<0.05
**MCV** [fL]											
Control	89.3 ± 3.9	-	0.316	89.9 ± 3.1	-	0.427	89.8 ± 2.0	-	0.732	0.43	0.626
Arg	92.1 ± 3.0	0.35		91.8 ± 2.8	0.559		93.5 ± 0.3	<0.05		0.647	0.278
IHE	85.5 ± 3.4	0.163		84.8 ± 2.4	<0.05		83.8 ± 1.9	<0.001		0.328	0.067
Arg/IHE	88.0 ± 3.2	0.842		87.8 ± 3.1	0.406		85.0 ± 3.5	<0.001		0.681	<0.05
**MCH** [pg/RBC]											
Control	28.4 ± 1.0	-	0.393	28.5 ± 1.5	-	0.311	30.5 ± 0.9	-	0.643	1	<0.001
Arg	28.7 ± 1.5	0.963		28.6 ± 0.9	1		32.0 ± 0.6	<0.05		1	<0.001
IHE	28.8 ± 1.4	0.934		29.7 ± 1.9	0.377		28.5 ± 1.0	<0.01		0.079	0.451
Arg/IHE	30.7 ± 1.2	<0.01		30.6 ± 1.1	<0.05		28.6 ± 1.5	<0.01		0.681	<0.05
**MCHC** [g/dL]											
Control	31.7 ± 0.9	-	0.826	31.7 ± 1.1	-	0.863	33.7 ± 0.4	-	0.158	0.968	<0.01
Arg	31.0 ± 0.6	0.241		31.2 ± 0.3	0.522		34.2 ± 0.3	0.221		0.359	<0.05
IHE	33.5 ± 0.2	<0.001		35.1 ± 0.9	<0.001		33.7 ± 0.6	1		<0.001	0.336
Arg/IHE	34.8 ± 0.8	<0.001		34.9 ± 0.3	<0.001		33.8 ± 0.5	1		0.155	<0.05
**RDW** [%]											
Control	15,2 ± 1,7	-	0.525	15.0 ± 1.3	-	0.594	15,0 ± 0.9	-	0.85	0.797	0.743
Arg	14,7 ± 0.4	0.839		14.9 ± 0.6	0.973		14.5 ± 0.0	0.282		0.352	0.26
IHE	12.4 ± 0.4	<0.001		12.4 ± 0.4	<0.001		12.4 ± 0.3	<0.001		0.741	0.618
Arg/IHE	15.3 ± 0.9	0.991		14.9 ± 0.6	0.975		12.3 ± 0.4	<0.001		0.214	<0.001
**EPO** [mIU/mL]											
Control	3.25 ± 0.88	-	0.618	4.3 ± 1.37	-	0.466	4.84 ± 1.17	-	0.654	<0.01	<0.01
Arg	3.51 ± 0.42	0.922		6.08 ± 1.04	<0.05		4.40 ± 1.68	0.994		0.618	<0.05
IHE	3.12 ± 0.86	0.99		6.57 ± 0.71	<0.01		9.70 ± 2.32	<0.001		<0.01	<0.001
Arg/IHE	4.62 ± 0.8	<0.01		6.49 ± 0.73	<0.01		8.13 ± 1.62	<0.001		<0.001	<0.01
**WBC** [10^3^/µL]											
Control	5.9 ± 1.0	-	0.149	6.7 ± 0.7	-	0.25	6.9 ± 0.4	-	0.173	0.066	<0.05
Arg	5.6 ± 0.1	0.774		5.9 ± 0.8	0.3		6.5 ± 0.8	0.957		0.281	<0.05
IHE	5.0 ± 0.3	0.148		5.3 ± 0.7	<0.05		6.1 ± 0.3	0.619		0.114	<0.01
Arg/IHE	5.5 ± 1.0	0.654		6.2 ± 1.2	0.699		7.6 ± 2.2	0.591		0.322	<0.05

**Abbreviations**: Arg, arginine supplementation; IHE, intermittent hypoxic exposure; Arg/IHE, arginine supplementation and intermittent hypoxic exposure; HB, haemoglobin; RBC, red blood cells; RET, reticulocytes; HCT, haematocrit; MCV, mean cell volume; MCH, mean corpuscular haemoglobin; MCHC, mean corpuscular haemoglobin concentration; RDW, red cell distribution width; EPO, erythropoietin; WBC, white blood cell count; η^2^ is a measure of effect size. Data in columns whose names begin with “Control” show the P-values of the Tukey’s post-hoc tests of the univariate one-way ANOVA examining, separately, each dependent variable. The last two columns show the P-values of the t-Student test or the Wilcoxon nonparametric test (if the normality assumption is violated).

**Table 6:** Lipoprotein–lipid profile.

	1st Day of Camp			7th Day of Camp			14th Day of Camp			1st Day vs. 7th Day	1st Day vs. 14th Day
	mean ± SD	Control vs. Arg, IHE or Arg/IHE	*η* ^2^	mean ± SD	Control vs. Arg, IHE or Arg/IHE	*η* ^2^	mean ± SD	Control vs. Arg, IHE or Arg/IHE	*η* ^2^		
**TG** [mg/dL]											
Control	97 ± 34	-	0.057	83 ± 24	-	0.231	118 ± 42	-	0.359	<0.05	0.31
Arg	85 ± 24	0.925		76 ± 12	0.951		64 ± 4	<0.01		0.331	0.057
IHE	111 ± 41	0.898		112 ± 18	0.138		122 ± 27	0.995		0.975	0.357
Arg/IHE	105 ± 50	0.969		78 ± 35	0.978		111 ± 32	0.964		0.06	0.764
**TC** [mg/dL]											
Control	196 ± 25	-	0.143	189 ± 38	-	0.315	175 ± 19	-	0.306	0.323	<0.05
Arg	160 ± 47	0.238		138 ± 25	<0.05		148 ± 7	0.123		0.097	0.522
IHE	188 ± 38	0.976		161 ± 31	0.399		163 ± 27	0.789		<0.05	<0.05
Arg/IHE	196 ± 41	1		189 ± 34	1		188 ± 30	0.601		0.399	0.359
**LDL** [mg/dL]											
Control	118 ± 27	-	0.149	121 ± 33	-	0.173	111 ± 18	-	0.059	0.707	0.232
Arg	93 ± 32	0.395		77 ± 17	0.555		97 ± 10	0.692		0.094	0.698
IHE	112 ± 38	0.985		103 ± 38	0.956		105 ± 37	0.963		0.406	0.386
Arg/IHE	128 ± 35	0.913		157 ± 117	0.664		112 ± 30	0.999		0.432	0.129
**HDL** [mg/dL]											
Control	64 ± 43	-	0.094	51 ± 11	-	0.114	53 ± 10	-	0.188	0.234	0.76
Arg	50 ± 13	0.716		45 ± 9	0.675		53 ± 5	0.996		0.182	0.47
IHE	45 ± 10	0.498		56 ± 12	0.787		48 ± 7	0.667		<0.01	<0.05
Arg/IHE	47 ± 7	0.493		49 ± 7	0.998		45 ± 8	0.176		<0.05	0.641
**Non-HDL** [mg/dL]											
Control	132 ± 44	-	0.126	138 ± 36	-	0.312	122 ± 15	-	0.385	1	0.454
Arg	110 ± 35	1		92 ± 17	<0.05		95 ± 2	0.105		0.084	0.298
IHE	143 ± 42	1		106 ± 39	0.234		116 ± 28	0.951		<0.05	<0.001
Arg/IHE	149 ± 40	1		139 ± 31	1		143 ± 33	0.215		0.205	0.313

**Abbreviations**: Arg, arginine supplementation; IHE, intermittent hypoxic exposure; Arg/IHE, arginine supplementation and intermittent hypoxic exposure; TG, triglycerides; TC, total cholesterol; LDL, low-density lipoproteins; HDL, high-density lipoproteins; non-HDL, cholesterol calculated by subtracting the HDL value from a TC; η^2^ is a measure of effect size. Data in columns whose names begin with “Control” show the P-values of the Tukey’s post-hoc tests of the univariate one-way ANOVA examining, separately, each dependent variable. The last two columns show the *P*-values of the t-Student test or the Wilcoxon nonparametric test (if the normality assumption is violated).
